# Which assessment for the carriers: the cardiac view

**DOI:** 10.1186/1750-1172-10-S1-I3

**Published:** 2015-11-02

**Authors:** Arnt V Kristen

**Affiliations:** 1 Amylodosis Center, Department of Cardiology, University of Heidelberg, Germany

## 

Cardiac manifestation is common in transthyretin-related (ATTR) amyloidosis. However, the age of onset of first symptoms ranges widely. Moreover, it differs between the individual mutations.

Potential treatment options to disrupt amyloid fibrils are lacking and cardiac manifestation is associated with poor prognosis. Thus, early identification of patients at risk for development of systemic ATTR amyloidosis is crucial to avoid deterioration of organ function and maintain quality of life. Predictive moleculargenetic testing of family members is the initial step to identify a patient at risk for hereditary ATTR amyloidosis. However, incomplete penetrance and high variability of age of onset of disease complicates the diagnostic strategy. Thus, regular screening is required for early identification of the disease. However, the question arises what diagnostic tools should be used for assessment of asymptomatic carriers of a TTR gene variant.

Although endomyocardial biopsy has the highest sensitivity and specificity for detection of cardiac amyloidosis it is not capable for longitudinal evaluation in asymptomatic carriers. Non-invasive strategies are required to allow frequent testings with time intervals ranging between 6 and 24 months. ECG, echocardiography, cardiac magnetic resonance imaging, skeletal scintigraphy, and cardiac biomarkers are potential tools for assessment. In general, routine assessment should be based on ECG and echocardiography. Precise screening of ECG for any abnormalities including conduction disturbances, QRS or T-wave abnormalities is mandatory as abnormal findings are present in almost all patients with cardiac ATTR amyloidosis. Echocardiography assessment should include evaluation of diastolic function, longitudinal impairment, as well as speckle tracking. Cardiac MRI has become the gold standard for evaluation of cardiac morphology and function. Moreover, application of gadolinium allows characterization of the myocardium by a characteristic pattern of late gadolinium enhancement in cardiac amyloidosis. Despite high resolution images and excellent reproducibility, this technique is time consuming, cost intensive, and ultimately ira availability is limited to large centers. Recently, native cardiac T1 mapping has been reported to abnormal early during the clinical course.

Cardiac retention of bone tracers has been reported to be specific for detection of ATTR amyloid. However, the clinical use for early identification remains debatable. Finally, longitudinal assessment is limited due to radioactivity. Cardiac biomarkers, including brain-natriuretic peptides and troponin T are sensitive indicators of myocardial stress and injury and appear to be an easy and useful tools for work-up of carriers.

Due to lack of potential treatment options to disrupt amyloid fibrils the main goal in carriers of transthyretin variants is to dectect manifest amyloid disease as early as possible. From the cardiological point of view carriers of TTR variants should routinely be assessed by ECG, echocardiography with speckle tracking, and cardiac biomarkers. If available cardiac MRI with mapping techniques appears to be of additional benefit.

